# *TaYS1A*, a Yellow Stripe-Like Transporter Gene, Is Required for Wheat Resistance to *Puccinia striiformis* f. sp. *Tritici*

**DOI:** 10.3390/genes11121452

**Published:** 2020-12-03

**Authors:** Md Ashraful Islam, Jia Guo, Huan Peng, Shuxin Tian, Xingxuan Bai, Haochuan Zhu, Zhensheng Kang, Jun Guo

**Affiliations:** State Key Laboratory of Crop Stress Biology for Arid Areas, College of Plant Protection, Northwest A and F University, Yangling 712100, China; a.islam160@nwafu.edu.cn (M.A.I.); guojia1889@nwafu.edu.cn (J.G.); penghuan@nwafu.edu.cn (H.P.); tianshuxin@nwafu.edu.cn (S.T.); baixingxuan@nwafu.edu.cn (X.B.); zhuhaochuan@nwafu.edu.cn (H.Z.); guojunwgq@nwsuaf.edu.cn (J.G.)

**Keywords:** wheat, yellow stripe-like transporter, salicylic acid, *Puccinia striiformis* f. sp. *tritici*, resistance, VIGS

## Abstract

Yellow stripe-like (YSL) transporters are required for the transportation of metal-phytosiderophores and are structurally related to metal-nicotianamine complexes. Some studies also reported the involvement of YSL transporters in pathogen-induced defense. However, the molecular mechanisms of *YSL* genes involved in biotic stress responses are still not clear, especially in cereal crops. This study aimed to functionally characterize *TaYS1A* during the interaction of wheat and *Puccinia striiformis* f. sp. *tritici* (*Pst*), the causal agent of stripe rust disease. TaYS1A was localized in the cell membrane of wheat protoplasts and *Nicotiana benthamiana* cells. *TaYS1A* was significantly up-regulated in wheat leaves after being infected with the avirulent *Pst* isolate CYR23 and after treatment with salicylic acid (SA). Silencing of *TaYS1A* by the virus-induced gene silencing method enhanced the susceptibility of wheat to *Pst* accompanied by reducing the accumulation of SA and H_2_O_2_ and down-regulating the transcriptions of *TaPR1* and *TaPR2*. In addition, TaYS1A was found to interact with TaNH2, a homolog of OsNH2, by yeast-two-hybrid assay, and silencing of *TaYS1A* diminished the expression of *TaNH2*. Our findings suggested the existence of positive regulation of *TaYS1A* in providing resistance against *Pst* by modulating SA-induced signaling and offered new insight into the biological role of *YSL* in wheat against pathogens.

## 1. Introduction

*Pst*, causing wheat stripe rust, is a major challenge to global wheat production. One of the most economical and productive strategies for stripe rust management is to develop resistant wheat cultivars. Dissection of the molecular interaction pathways between wheat and *Pst* would promote the appropriate use of resistance genes for varietal improvement. Usually, plants produce a sequence of immune responses to defend against pathogen invasion, such as ion fluxes through the plasma membrane, increased intracellular Ca^2+^ concentration, accumulation of reactive oxygen species (ROS), biosynthesis of salicylic acid (SA), fortification of the cell-wall, a hypersensitive response (HR), the formation of localized cell wall appositions (CWAs or papillae), among others [1,2,3,4]. Among those, CWAs is one of the common innate or basal defense response against pathogens infection by a burst of ROS and pathogenesis-related (PR) protein production. Beyond the establishment of CWAs, ROS are also involved in several plant signaling regulations including local and systemic signaling essential for plant immunity [4,5,6]. Salicylic acid (SA) also was shown to be a crucial regulator of multiple stages of immunity among these signaling systems, protecting plants from a broad variety of microorganisms by mitigating local and systemic immune responses. SA signaling through NPR1 (non-expresser of pathogenesis-related protein 1) is essential for the development of systemic acquired resistance (SAR) [7,8]. In rice, over-expression of NH1/NH3 (NPR1 homologs) exhibited increased resistance to *Magnaporthe oryzae* and *Xoo*, suggesting its positive roles in host resistance against pathogens [9,10]. Liu, et al. [11] reported that an OsNH2 homolog TaNH2, was induced by *Pst* infection and SA treatment and was required for wheat resistance to *Pst*. However, research on the functions of *NPR1* homologous genes in wheat is still quite limited.

Iron (Fe) is one of the most important micronutrients required for different physiological processes of plants, e.g., respiration, chlorophyll biosynthesis, photosynthesis, and nitrogen assimilation [12,13]. In addition to involvement in physiological processes, Fe is readily involved in one electron reduction-oxidation (redox) reactions to catalyze the formation of ROS through the Fenton reaction [14,15]. Maize in Fe-deficient condition was unable to produce ROS at the site of *Colletotrichum* infection, which increased susceptibility to this fungus [16]. In turn, ROS production, especially resistance-linked hydrogen peroxide (H_2_O_2_), is dependent on the accumulation of redox-active ferric iron ions (Fe^3+^) in cereal crops [16,17,18]. Reactive Fe^3+^ accumulated at cell wall appositions to induce oxidative burst and intracellular depletion of Fe provoked by pathogen infection; this condition led to the induction of pathogenesis-related genes along with H_2_O_2_ [18]. Immediately after pathogen invasion, ROS accumulation begins, which has been linked with the generation of superoxide in apoplast to induce a signal for further immune response by Fe-containing transmembrane NADPH oxidases but that is impermeable to plant cell membrane. The generation of membrane-permeable H_2_O_2_ can take place from the conversion of superoxide by superoxide dismutases; in some of these Fe has a function as a cofactor [19,20,21,22]. Moreover, some recent studies in rice revealed an important function of Fe as a regulator of HR cell death during effector-triggered immunity (ETI) through a ferroptosis mechanism in response to avirulent race of rice blast pathogens [23,24]. In *Arabidopsis*, *frd3* mutant disrupted the localization of Fe in the cell wall, triggering biotic stress response through ROS production [25]. Thus, complex regulatory mechanisms have been involved during plant-pathogens interactions that harmonize Fe acquisition and homeostasis. However, plant species possess two classes of strategies to acquire a sufficient amount of Fe from the soil in a regulated manner. Nongraminaceous plants used strategy I to decrease ferric chelates at the root surface and absorb ferrous ions across the root plasma membrane via iron transporters. In grasses, yellow stripe-like (YSL) transporters take up Fe-PS (phytosiderophores) complexes from the soil and translocate throughout the plant by strategy II [26,27,28,29,30].

YSLs belong to the oligopeptide transporter (OPT) family, one of the major groups of membrane-bound integral proteins that are involved in the transfer, detoxification, or remobilization of metals, relying on the nutrient levels in the plant and its position of action [31,32,33]. Long-distance transport in shoots/leaves is carried out via phloem, in which YSLs have also been reported to be involved in nutrient remobilization of storage tissue [34,35]. In addition to metal transportation, YSLs have been found to take part in the defense against pathogens. In *Arabidopsis*, *YSL3* was found to be strongly expressed in the *siz1* mutant, and the plant displayed elevated levels of SA and SA glucoside. Elevation of SA in siz1 plants triggered transcription of pathogenesis-related (*PR*) genes, improving resistance to pathogenic bacteria [36,37]. It was also reported that the transcription of *AtYSL3* could be triggered by SA through an *NPR1*-dependent pathway, which positively regulates the basal resistance [38]. *NPR1* in *Arabidopsis*, is a receptor of SA and is a transcriptional coregulator of SA-dependent defense-related gene activation [39,40]. *NPR1* modulates the transcript of almost 99% of SA-responsive genes, indicating its importance in SA signaling pathways and transcriptional reprogramming during plant defense. As transcriptional coactivators, *NPR1* monomers interact with TGA transcription factors, which bind the promoter of *PR* genes, to activate the expression of *PR* genes [41,42]. Thus, it is reasonable to assume that in presence of SA, *NPR1* regulates the activation of *YSLs* and the metal homeostasis function of *YSL* genes may induce ROS mediated HR and defense-related genes to establish plant immunity during pathogen infection. Nevertheless, in wheat, some studies on YSLs have been carried out and the functions of YSLs in wheat defense against *Pst* are still unknown.

In the present investigation, we carried out *TaYS1A* gene isolation and characterized its roles during wheat-*Pst* interactions. We analyzed the transcript level of *TaYS1A* in *Pst*-inoculated wheat seedlings and hormone-treated plants and observed the subcellular localization of TaYS1A. We found that suppression of *TaYS1A* transcription enhances the susceptibility of wheat to *Pst*. Through yeast two hybrid assays and silencing of *TaYS1A*, we also found that TaYS1A interacted with TaNH2, an *Arabidopsis* NPR3/4 orthologue. Our results suggest that *TaYS1A* plays a positive role in SA-dependent wheat resistance to the stripe rust fungus.

## 2. Materials and Methods

### 2.1. Plant Materials, Fungal Isolates and Treatments

Chinese wheat cultivar, Suwon11(Su11), *N. benthamiana*, and *Pst* isolates CYR23 and CYR31 were used as the study materials. Wheat (*Triticum aestivum* L.) variety Suwon11 (AUS-22519) originated from the Seuseun Agricultural Experiment Station (Sariwon, Korea) was registered in the Australian Winter Cereal Collection, Tamworth, Australia. Su11 carrying a stripe rust resistance gene *YrSu*, produces an incompatible interaction with CYR23 and compatible interaction with CYR31 [43]. Maintenance of wheat seedlings growth and inoculation was performed as previously done by Kang, et al. [44]. For RNA extraction first leaves of CYR23, CYR31, or sterile distilled water (mock control) inoculated wheat seedlings were collected at 0, 6, 12, 18, 24, 36, 48, 72, and 120 hpi. In the case of hormone treatments, wheat seedlings were sprayed with 10 mM salicylic acid (SA), 100 mM abscisic acid (ABA), 100 mM methyljasmonate (MeJA), and 100 mM ethepon (ETH). The solvent 0.1% (*v*/*v*) ethanol was applied to treat mock control plants and then sampled at 0, 0.5, 1, 2, 12, 24 and 48 hpt for RNA extraction. Tissue-specific expression analysis was performed on root, stem, and leaves of 12 days wheat seedlings. To preserve the plant samples for further RNA isolation, they were instantly frozen in liquid N_2_ followed by storage at −80 °C. For every treatment, three independent biological replications were included.

### 2.2. RNA Extraction and qRT-PCR

For RNA extraction, the Quick RNA isolation Kit (Huayueyang Biotechnology, Beijing, China) was used following the manufacturer’s protocols. DNA contamination was eliminated through DNase I treatment. A 3-µg aliquot of the total RNA of each sample was used for cDNA synthesis with the RevertAid First Strand cDNA Synthesis Kit (Thermo Scientific, Waltham, MA, USA). *TaYS1A* expression analysis was conducted by qRT-PCR assay and presented as the *TaYS1A* transcript level in both virulent/avirulent *Pst* inoculated and chemical treated wheat leaves compared to mock control wheat leaves. Specific primers were designed for qRT-PCR analysis (Table S1), as suggested previously [45]. Threshold values (CT) were obtained with the ABI PRISM 7500 system (Applied Biosystems, Foster City, CA, USA). Then, using the comparative 2^−ΔΔCT^ method [46], the transcription level of *TaYS1A* was quantified. The *Elongation factor 1 (TaEF-1α)* was used to normalize the data (GenBank accession no.Q03033) [47] (Table S1). All experiments were replicated three times.

### 2.3. Sequence Analysis of TaYS1A

*TaYS1A* was amplified from CYR23-inoculated Su11 cDNA template with specific primers (Table S1). Sequencing of *TaYS1A* was done by purifying the PCR DNA and inserted into the pGEM-T Easy vector (Promega, Madison, WI, USA). The copies of *TaYS1A* and other related sequences were obtained from the Ensemble Plant database. Blast programs in NCBI (https://www.ncbi.nlm.nih.gov/) were used for analyzing sequences. ORF Finder (https://www.ncbi.nlm.nih.gov/orffinder/), NCBI Nucleotide Blast, and the Protein Blast programs (https://www.ncbi.nlm.nih.gov/) were used for the analyses of cDNA sequences.

### 2.4. Subcellular Localization of TaYS1A Protein

A pCaMV35S:TaYS1A-GFP fusion plasmid was generated for investigating the subcellular localization in wheat protoplasts. Wheat protoplasts were extracted from the mesophyll tissue of ten-day-old wheat seedlings as described [48]. The recombinant construct pCaMV35S:TaYS1A-GFP or pCaMV35S: GFP was transferred into wheat protoplasts by Polyethylene glycol (PEG)-calcium transfection [49]. For further confirmation of TaYS1A localization, we used *Agrobacterium* infiltration assay in *N. benthamiana. Agrobacterium* infiltration results in a large number of transformed cells within an intact tissue *N. benthamiana* that can easily be identified and analyzed at the subcellular level within a short period [50]. The recombinant pCAMBIA1302:TaYS1A-GFP fusion was constructed using *Avr*II and *Spe*I restriction sites and transformed into the *A. tumefaciens* strain GV3101 by electroporation. Infiltration of *N. benthamiana* leaves was done following the procedure described by Cheng, et al. [51]. Infiltrated plants were maintained for 2–3 days at 25 °C with a light cycle of 16 h light and 8 h dark and then tissue samples were collected to observe GFP autofluorescence. An Olympus FV1000 confocal laser microscope with a 488 nm filter was used to image GFP autofluorescence.

### 2.5. BSMV-Mediated VIGS of TaYS1A

Two specific cDNA fragments of *TaYS1A* (Figure S1) were designed and analyzed for VIGS. The fragments were obtained with *Not*I and *Pac*I restriction sites by reverse transcription PCR and cloned into BSMV: γ to modify the original barley stripe mosaic virus (BSMV), which is designated as BSMV:TaYS1A-1/2as [52]. No similarity in the fragments was found with any other wheat genes in the BLAST analyses (http://blast.ncbi.nlm.nih.gov/Blast/), indicating the specificity of the selected segments. Capped in vitro transcripts were obtained with the RiboMAX TM Large-Scale RNA Production System-T7 (Promega) and the Ribo m^7^G Cap Analog (Promega) following the manufacturer’s protocol. The second leaves of two-leaf-stage wheat seedlings were mechanically rubbed with BSMV constructs and incubated for 24 h under dark and humid conditions. After incubation, the inoculated plants were transferred to a growth chamber maintained at 25 ± 2 °C and a 16 h photoperiod. Mock plants were rubbed with 1 × Fes. Ten days after virus inoculation, the fourth leaves of wheat were inoculated with freshly harvested *Pst* isolates CYR23 or CYR31 urediniospores. The inoculated leaves were sampled at 0, 24, 48, and 120 hpi for RNA isolation and histological observations. The relative expression of *TaYS1A*, the expression level of two pathogenesis-related (PR) genes (*TaPR1* and *TaPR2*), and ROS-related genes were analyzed by qRT-PCR assay. Wheat phenotypes after *Pst* inoculation were examined based on the McNeal measurements scale [53] and photographed at 14 dpi. Fungal biomass was analyzed by qRT-PCR. Genomic DNA was extracted using the CTAB method from samples collected at 14 dpi after infected with *Pst* and a standard curve was generated by the plasmid carried the fragment of *PsEF1* and *TaEF1* as previously described [54,55]. All experiments were conducted in triplicate and 50 plants were used for each fragment at each time.

### 2.6. Histology of Host Response and Fungal Growth

The samples of *TaYS1A*-silenced plants were stained for histological observation. For the detection of H_2_O_2_ accumulation, infected leaves were stained with 3,3′- diaminobenzidine (DAB, Amresco, Solon, OH, USA) and then observed microscopically [56]. For visualizing pathogen structures, wheat germ agglutinin (WGA) conjugated to Alexa-488 (Invitrogen, Carlsbad, CA, USA), which specifically stains *Pst*, was used as described previously [57]. The site where an appressorium had formed over a stoma was considered to be a successful penetration site of *Pst*. The accumulation of H_2_O_2_, necrotic areas, hyphal length, haustoria mother cell, hyphal branches, and infection areas were observed under ultraviolet light with a microscope (Olympus Corporation, Tokyo, Japan), and the DP-BSW software (Olympus Corporation, Tokyo, Japan) was used to calculate length and area. A minimum of 50 infection sites were examined on each of five randomly selected leaf segments for each treatment. The experiment included three independent biological replications.

### 2.7. SA Analysis

For SA quantification, about 200 mg of fresh leaves were ground in liquid N_2_ and extracted with 750 µL of MeOH^−^:H_2_O^−^:HOAc (90:9:1, *v/v/v*). The analysis was done by HPLC-MS (API 2000; AB SCIEX, Framingham, MA, USA). All procedures and analyses were carried out with three biological replications according to the protocol used by Segarra, et al. [58].

### 2.8. Yeast Two-Hybrid Assay

The recombinant pGBKT7-TaYS1A vector was constructed by inserting the coding sequence of *TaYS1A* with primers TaYS1A-BD-F/R and the recombinant pGADT7-TaNH2 vector was constructed by inserting the coding sequence of *TaNH2* with primers TaNH2-AD-F/R (Table S1). For interaction assay, pGBKT7-TaYS1A and pGADT7-TaNH2 were co-transformed into the yeast strain AH109 by the lithium acetate method following the Yeast Protocols Handbook (Clontech) and plated on the SD/-Trp-Leu or SD/-Trp-Leu-His selection medium. We selected colonies from SD/-Trp-Leu-His and plated them on SD/-Trp-Leu-His-Ade medium again for further selection. The interaction was confirmed by plating on SD/-Trp-Leu-His-Ade medium containing X-α-gal. The combination of pGBKT7-TaCIPK10 and pGADT7-TaNH2 was used as a positive control [11] and the combination of pGBKT7-TaYS1A and pGADT7 empty vector was used as a negative control.

### 2.9. Statistical Analysis

Microsoft Excel was used to analyze the mean values and standard errors. The significant differences between controls and treatments or between time points were calculated by the Student’s *t*-test using SPSS 23 (SPSS Inc., Chicago, IL, USA).

## 3. Results

### 3.1. TaYS1A Expression is Induced upon Avirulent Pst Infection and SA Treatment

**Figure 1:**
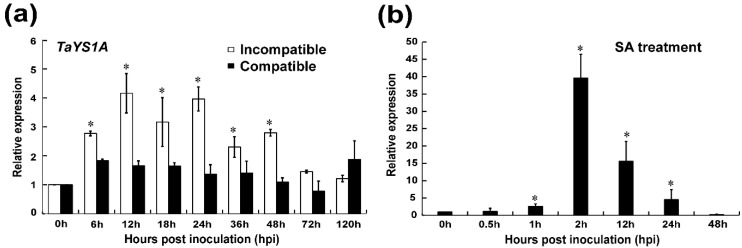
Analysis of *TaYS1A* expression in *Puccinia striiformis* f. sp. *tritici (Pst*) inoculated and salicylic acid (SA) treated wheat leaves. (**a**) *Pst* isolates CRY23 and CRY31 inoculated wheat leaves were sampled at various times, representing different stages of *Pst* infection. The mock was inoculated at 0 hpi. (**b**) Endogenous SA induces *TaYS1A* transcription. Wheat leaves sprayed with 10 mM SA and sampled at 0, 0.5, 1, 2, 12, 24, and 48-h post treatment (hpt). The comparative threshold (2^−ΔΔCT^) approach was used to measure the relative transcript levels of *TaYS1A*. Data normalized with the transcription of wheat elongation factor, *TaEF-1α*, and visualized as fold change compared to control at 0h. Each data point represents a mean ± the standard error of three independent biological repetitions. Asterisks denote significant difference (*p* < 0.05) from 0 hpi/hpt by the Student’s *t*-test.

Recently, Kumar, et al. [59] identified a total of 26 putative wheat *YSL* genes. The present study is principally focused on the wheat *YSL* gene, *TaYS1A*. We obtained the cDNA sequence by RT-PCR from wheat cultivar Su11. We found *TaYS1A* in the same clade with *AtYSL3* and its closest homolog *HvYS1* in barley (Figure S1). The full-length of *TaYS1A* (accession number: TraesCS6B02G283800) encodes a protein containing 678 amino acids with a molecular weight of 74.56 kDa and an isoelectric point (pI) 9.15. BlastN search in the hexaploid wheat genome database identified three copies of *TaYS1A* with 98.97% nucleotide coding sequence similarities. Therefore, considering high nucleotide coding sequence identity, *TaYS1A* in chromosome 6B was selected as a representative of three copies for further study. To investigate the role of *TaYS1A*, we further analyzed relative transcript profiles of *TaYS1A* in *Pst* inoculated and mock plants at different time points by qRT-PCR. The transcription of *TaYS1A* was significantly up-regulated at 6 to 48 h post-inoculation (hpi) and attained a peak of approximately 4.1-fold at 12 hpi in wheat leaves infected by CYR23 isolate over the control plants (Figure 1a).

Previous studies demonstrated that the expression of TaYS1A protein was tissue-specific in higher plants. We analyzed *TaYS1A* transcripts from various wheat tissues by qRT-PCR analysis to examine the tissue-specific expression in wheat. *TaYS1A* expression was detected in all tissues examined (root, stem, and leaf). The *TaYS1A* transcripts were significantly higher in roots and leaves than stems tissue (Figure S2a). To explore the roles of *TaYS1A* in hormone perception and signaling, we treated wheat seedlings with different phytohormones, including ABA, SA, ethylene (ET), and jasmonic acid (JA). After treating with SA, the expression of *TaYS1A* was significantly up-regulated at 1 h after treatment and attained a peak of approximately 39-fold at 2 h post-treatment (Figure 1b). When treated with MeJA, the transcription of *TaYS1A* was significantly downregulated at 6 to 24 h post-treatment (Figure S2b).

Since the *TaYS1A* expressions were significantly different in the incompatible interaction, SA-treatment, and MeJA treatment compared to the controls, we hypothesized that *TaYS1A* could be one of the positive regulators of wheat resistance to *Pst* infections. Further experiments were conducted accordingly to examine this hypothesis.

### 3.2. TaYS1A Encodes a Plasma Membrane-Targeted Protein

**Figure 2:**
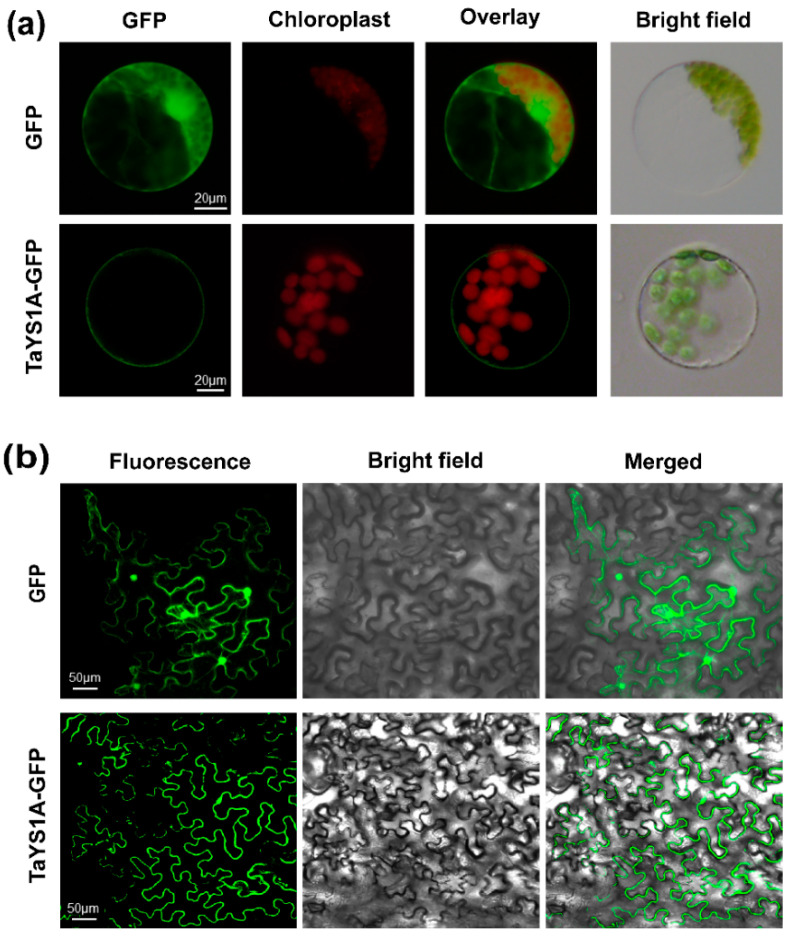
TaYS1A protein subcellular localization assays. (**a**) The PEG-mediated transformation was used to overexpress Green Fluorescent Protein (GFP) or TaYS1A-GFP fused proteins in wheat protoplasts. (**b**) GFP or TaYS1A-GFP construct were transformed into *A. tumefaciens* and then infiltrated into *N. bentamiana* leaves. The GFP autofluorescence was imaged using an Olympus FV1000 confocal microscope with a 488 nm filter.

In wheat, Kumar, et al. [59] predicted that most of the YSL proteins would be localized to the plasma membrane, with the remaining YSLs in the cytoplasm, chloroplast, vacuole, and mitochondria. To examine the subcellular localization of TaYS1A, transient overexpression of recombinant TaYS1A-GFP was achieved in wheat mesophyll protoplasts and *N. benthamiana* leaves. Under the fluorescence microscope, cells transfected with the empty GFP (control) vector displayed fluorescence throughout the cell, including the nucleus, whereas in the wheat protoplasts and *N. benthamiana* cells overexpressing *TaYS1A-GFP*, we observed fluorescence only on the plasma membrane (Figure 2).

### 3.3. Transient Silencing of TaYS1A Enhances Wheat Susceptibility to Pst

**Figure 3:**
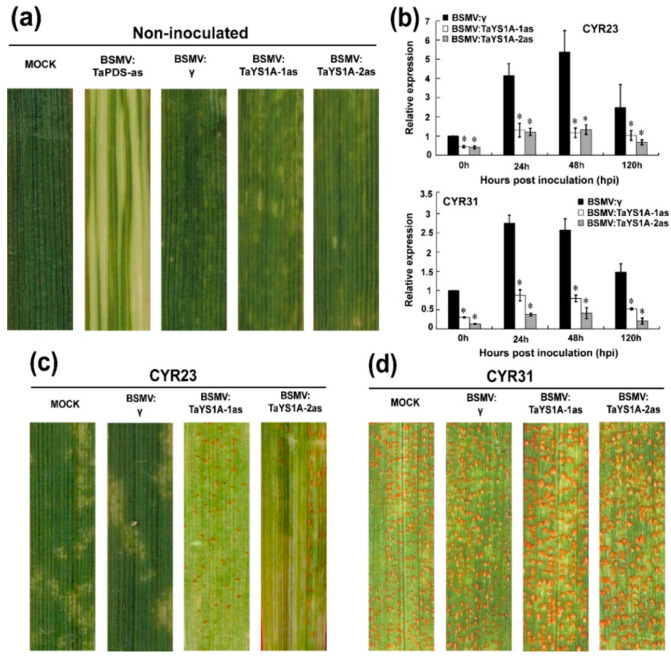
BSMV-mediated gene silencing (VIGS) of *TaYS1A* enhances wheat susceptibility to *Pst*. (**a**) At 10 dpi, chlorotic mosaic symptoms were observed on virus-inoculated leaves. Mock, second leaves of wheat seedlings were mechanically rubbed with 1 × Fes buffer. (**b**) Relative expression of *TaYS1A* in *TaYS1A*-silenced plants infected with the avirulent race CRY23 or virulent race CRY31, respectively. The comparative threshold (2^−ΔΔCT^) approach was used to measure the relative transcript levels of *TaYS1A*. Data normalized with the transcription of wheat elongation factor, *TaEF-1α*, and visualized as fold change compared to control at 0 h. BSMV:γ infected wheat leaves were used as a control. Values represent the mean ± standard errors of three independent biological samples. Asterisks designate significant differences (*p* < 0.05) from BSMV:γ by the Student’s *t*-test. (**c**,**d**) Photographs of the fourth leaves infected with urediniospores of CRY23 (**c**) and CRY31 (**d**) were taken at 14 dpi.

To determine the role of *TaYS1A* in the wheat resistant responses to *Pst*, we used BSMV-VIGS (virus-induced gene silencing), an effective and rapid gene-silencing method to analyze functions of the wheat and barley genes [60]. At ten-days post-inoculation, mosaic symptoms of the virus were observed in virus-inoculated plants, but further leaf growth was not affected. In contrast, BSMV:TaPDS-as inoculated plants gradually displayed photobleaching symptoms (Figure 3a), suggesting that the virus-induced gene silencing was successfully achieved. The *phytoene desaturase (PDS)* is required to protect plant chlorophyll from photo-bleaching and silencing of the *PDS* gene in monocot plants; lack of the *PDS* gene caused loss of chlorophyll pigmentation (photo-beaching) [52,61]. Thus, the fragment of wheat phytoene desaturase (*TaPDS*) was inserted into the BSMV vector to construct BSMV: TaPDS and was used as a positive visual control. qRT-PCR results revealed that the transcript levels of endogenous *TaYS1A* of the transformants were significantly reduced during incompatible and compatible interactions (Figure 3b). The copies of *TaYS1A* were highly identical, due to the expression level of individual copies which could not be analyzed by qRT-PCR (Figure S3). Remarkably, after inoculation with avirulent CYR23, all leaves expressed HR symptoms, whereas leaves pre-infected with BSMV:TaYS1A-1/2as were found to have few uredia of fungus around the HR. Control plants, BSMV:γ, and mock expressed only slight HR symptoms (Figure 3c). After inoculation with the virulent race CYR31, fungal growth was slightly increased on BSMV:TaYS1A-1/2as plants compared to the control BSMV:γ and compared to mock plants (Figure 3d).

**Figure 4:**
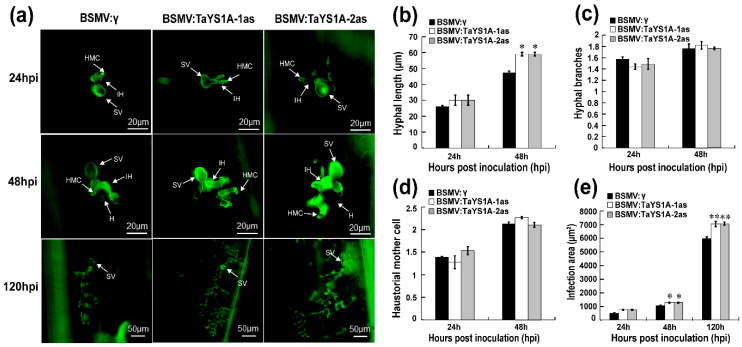
Histology of *Pst* avirulent race CYR23 in *TaYS1A*-silenced leaves. (**a**) Wheat germ agglutinin (WGA) was used to visualize fungal structures in wheat leaves infected with BSMV and *Pst* and the fungal structures were observed under an autofluorescence microscope. SV, sub-stomatal vesicle; HMC, haustorial mother cell; IH, infection hypha. H, haustoria. (**b**) Hyphal length is the average distance to the tip of the hypha from the intersection of the sub-stomatal vesicle and the hypha and was measured using DP-BSW (units in μm) program (Olympus Corporation, Tokyo, Japan). (**c**) The average number of hyphal branches was estimated for each infection site. (**d**) The average number of haustorial mother cells in each infection site was determined. (**e**) Infection area is the average of developing hyphae, and was measured using the DP-BSW program (units in μm^2^). All findings were derived from three biological repetitions and each replication considered 50 sites of infection. Asterisks (*p* < 0.05) or double asterisks (*p* < 0.01) designate significant differences compared to the control plants at the same time points, using the Student’s *t*-test.

We also microscopically examined *TaYS1A-*silenced leaves infected with CYR23 and CYR31 *Pst* isolates. At 48 hpi wheat leaves infected with CYR23 at hyphal length significantly increased in *TaYS1A-*silenced plants in relation to BSMV:γ infected plants, and the infected area significantly expanded in *TaYS1A-*silenced plants at 120 hpi (Figure 4a–e), indicating that the suppression of *TaYS1A* transcription increased host susceptibility during incompatible wheat and *Pst* interactions. During CYR31 infection, as compared to the control plants, hyphal length in *TaYS1A-*silenced plants also significantly increased at 48 hpi, and the infection area also significantly expanded at 120 hpi (Figure S4a–c). Taken collectively, our results suggest that the silencing of *TaYS1A* in wheat benefits the growth and development of *Pst*, and thus increases wheat susceptibility to both avirulent and virulent isolates of *Pst*.

### 3.4. H_2_O_2_ Accumulation and PR Transcription Are Affected in TaYS1A-Silenced Plants

**Figure 5:**
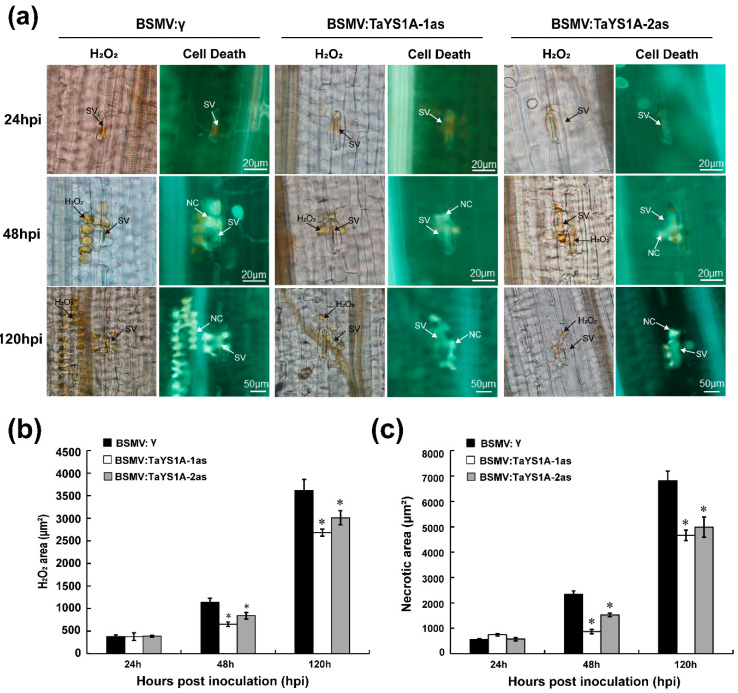
H_2_O_2_ accumulation in *TaYS1A-*silenced leaves was reduced when infected with *Pst* avirulent race CYR23. (**a**) Histological observation of wheat leaves inoculated with BSMV and infected with CYR23. Wheat leaves pre-infected with BSMV:γ or BSMV:TaYS1A-1/2as were then inoculated with CYR23. 3,3-diaminobenzidine (DAB) was used for H_2_O_2_ detection. The necrosis was detected by autofluorescence and was viewed under differential interference contrast optics. SV, sub-stomatal vesicle; NC, necrotic cell. (**b**) The amount of H_2_O_2_ production was calculated using DP-BSW tools by measuring the DAB-stained region at each infection site. (**c**) To calculate necrotic cell death, the fluorescence area was measured. Values denote the mean ± standard errors of three independent biological samples. Asterisks designate significant differences (*p* < 0.05) from BSMV:γ at same time points by the Student’s *t*-test.

We microscopically examined the *TaYS1A-*silenced leaves infected with CYR23 and CYR31 to explore why *TaYS1A-*silenced leaves were more susceptible to *Pst*. Our results revealed that the accumulation of H_2_O_2_ was significantly decreased in *TaYS1A-*silenced leaves as compared to the control leaves at 48 and 120 hpi during incompatible interactions (Figure 5a,b). Moreover, the necrotic cells per infection site of the *TaYS1A-*silenced leaves also decreased significantly at 48 and 120 hpi, compared to the control plant leaves (Figure 5a,c). These results indicate that increased susceptibility of wheat to *Pst* after silencing of *TaYS1A* seems to rely on ROS.

**Figure 6:**
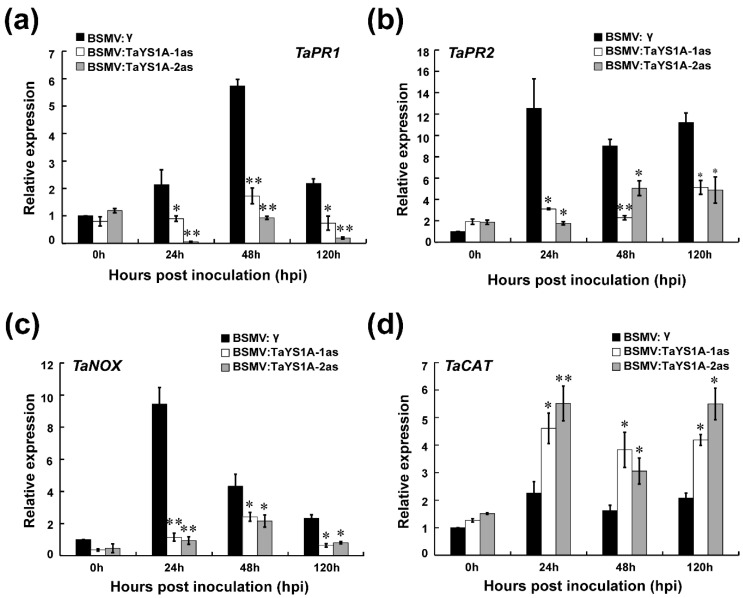
Relative expression of defense and reactive oxygen species (ROS) related marker genes in *TaYSIA-*silenced plants infected with *Pst* avirulent race CYR23 (**a**) The relative expression of *TaPR1* (pathogenesis-related gene 1). (**b**) The relative expression of *TaPR2* (*β*-1,3-glucanase). (**c**) The relative expression of *TaNOX* (NADPH oxidase) (**d**) The relative expression of *TaCAT* (catalase). The qRT-PCR was used to analyze the relative expression of all genes. The comparative threshold (2^−ΔΔCT^) approach was used to measure the relative transcript levels of *TaYS1A*. Data normalized with the transcription of wheat elongation factor, *TaEF-1α*, and visualized as fold change compared to control at 0h. BSMV:γ infected wheat leaves were used as a control. Values represent the mean ± standard errors of three independent biological samples. Asterisks (*p* < 0.05) or double asterisks (*p* < 0.01) designate significant differences from BSMV:γ by the Student’s *t*-test.

We further measured the expression of defense-related genes and ROS-related genes in *TaYS1A-*silenced plants after *Pst* inoculation. The transcript levels of *TaPR1*, *TaPR2*, and the ROS-generating gene, NADH-oxidase (*TaNOX*), were significantly diminished in *TaYS1A-*silenced plants relative to the control plants during incompatible interactions (Figure 6a–c). The transcription levels of the ROS-scavenging gene, *TaCAT*, were significantly up-regulated in *TaYS1A-*silenced plants inoculated with CYR23 (Figure 6d). When the *TaYS1A-*silenced plants were challenged by the virulent CRY31, transcript levels of *TaPR1* and *TaPR2* were also significantly diminished (Figure S5). Altogether, these results suggest that silencing of *TaYS1A* in wheat enhances *Pst* growth by restricting the transcription of PR proteins or H_2_O_2_ accumulation.

### 3.5. Fungal Biomass and SA Level in TaYS1A-Silenced Plants

**Figure 7:**
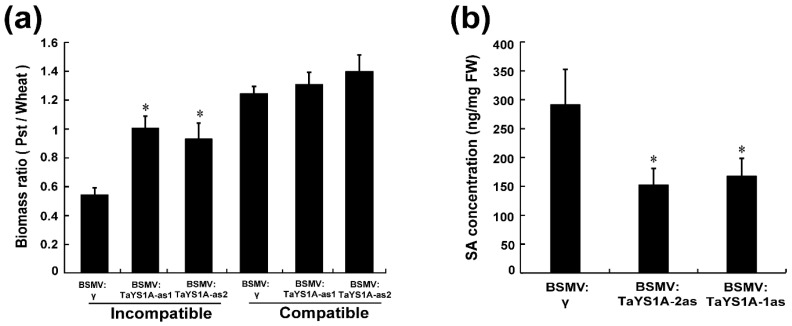
Fungal biomass and SA quantification in *TaYS1A*-silenced plants. (**a**) Relative fungal biomass was measured from the total genomic DNA of *TaYS1A-*silenced leaves infected with CYR23 or CYR31, which was sampled at 14 dpi. (**b**) SA concentration in *TaYS1A-*silenced leaves infected with CYR23, which was sampled at 14 dpi. Values denote the mean ± standard errors of three independent biological samples. Asterisks indicate significant differences from BSMV:γ using the Student’s *t*-test (*p* < 0.05).

To analyze the fungal biomass in *TaYS1A*-silenced leaves, total genomic DNA was extracted from the CYR23- and CYR31-inoculated leaves sampled at 14 days post-inoculation (dpi), and the biomass ratio was analyzed by qRT-PCR. The fungal biomass was found significantly increased in the *TaYS1A*-silenced leaves infected with CYR23 compared to the control plants (Figure 7a). In the *TaYS1A*-silenced leaves infected with CYR31, the fungal biomass was also slightly increased compared to the control plants.

Moreover, we measured the SA level in *TaYS1A*-silenced plants to determine whether the suppression of the *TaYS1A* gene affected the SA level and thereby led to the increased susceptibility to *Pst*. Our results showed that silencing of *TaYS1A* significantly reduced the endogenous SA concentration compared to control plants (Figure 7b).

### 3.6. TaYS1A Interacts with TaNH2

**Figure 8:**
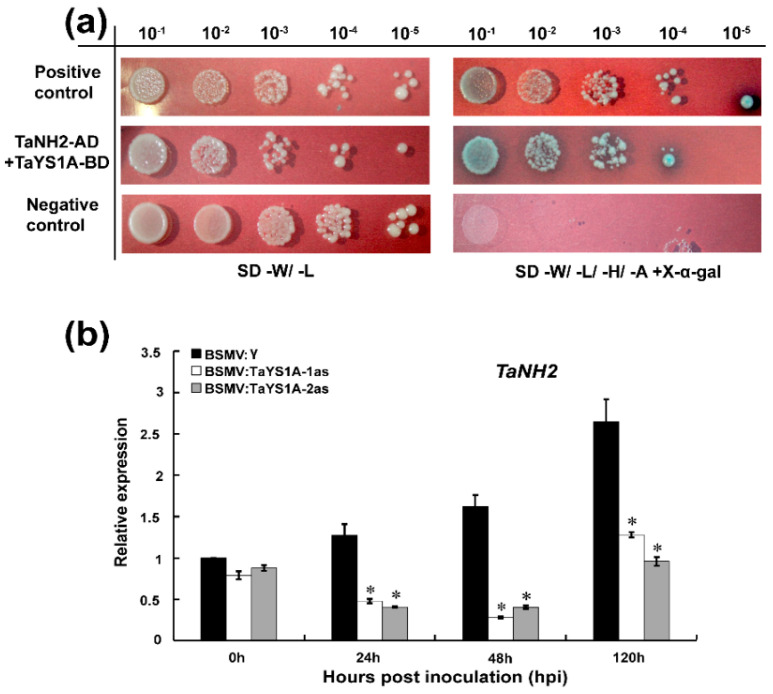
Interaction between TaYS1A and TaNH2. (**a**) Y2H interaction analysis between TaYS1A and TaNH2 in yeast. The constructed TaYS1A-BD interacts with TaNH2-AD in yeast. Cells of yeast strain AH109 containing the indicated pairs of plasmids were cultured on selective media SD-W/-L or SD- W/-L/-H/-A (SD/-Trp/-Leu or SD/-Trp/- Leu/-His/-Ade containing 20 μg/mL X-α-gal). Plates were photographed 3 dpi. SD, synthetic dropout growth medium. (**b**) Silencing of *TaYS1A* suppresses the transcription of *TaNH2*. Relative expression of *TaNH2* in *TaYS1A*-silenced leaves inoculated with CYR23 was determined by qRT-PCR. The qRT-PCR was used to analyze the relative expression of all genes. The comparative threshold (2^−ΔΔCT^) approach was used to measure the relative transcript levels of *TaYS1A*. Data normalized with the transcription of wheat elongation factor, *TaEF-1α*, and visualized as fold change compared to control at 0h. BSMV:γ infected wheat leaves were used as a control. Values represent the mean ± standard errors of three independent biological samples. Asterisks (*p* < 0.05) designate significant differences from BSMV:γ by the Student’s *t*-test.

Chen, et al. [38] reported that *YSL* genes function as downstream elements of SA signaling in an *NPR1-*dependent pathway. Based on this demonstration, we assumed that TaYS1A might directly interact with NPR1 or NPR1-homologous proteins. To explore our speculation, we selected TaNH2, an orthologue of AtNPR3/4, which showed a positive function in wheat resistance to *Pst* [11], and cloned it in pGADT7 vector. The constructs, pGBKT7-*TaYS1A*, and pGADT7-*TaNH2* plasmids were then co-transformed into the yeast strain AH109 cultured on the selection medium SD/-Trp/-Leu/-His/-Ade and X-α-gal. We found that TaYS1A strongly interacted with TaNH2 (Figure 8a).

To determine whether the silencing of *TaYS1A* affected the expression of *TaNH2*, we further analyzed the transcript level of *TaNH2* in *TaYS1A*-silenced plants by qRT-PCR. We found that the expression of *TaNH2* was significantly downregulated in the *TaYS1A*-silenced plants infected with CYR23 compared to the control plants (Figure 8b).

## 4. Discussion

The YSL-protein-family plays a key role in plants, particularly in the mobilization and long-distance transportation of micronutrients. YSL proteins were also found to be affected by the SA level and involved in the host defense against pathogens [38,62]. To date, the metal transportation ability of YSLs has been identified and characterized in different plant species, including *Arabidopsis*, rice, maize, and *Brachypodium* [63,64,65]. However, the biological functions of YSL proteins in pathogen induced defense are largely unknown, particularly in wheat defense against *Pst* infections. In our present study, we cloned a wheat *YSL* subfamily gene, *TaYS1A*, and characterized its molecular function during wheat-*Pst* interaction. The expression of *TaYS1A* was strongly induced when inoculated with *Pst* avirulent race CYR23 and SA treatment. Silencing of *TaYS1A* enhanced the susceptibility to both avirulent and virulent *Pst* races. The reduced expression of *PR-*genes and SA accumulation suggests that *TaYS1A* exhibits a positive role in wheat resistance to *Pst*.

YSLs are members of the OPT family, representing the largest subgroup of transmembrane proteins that are mainly involved in the transfer of a wide variety of substrates [31]. In wheat, a total of 26 genes of the *YSL* subfamily were previously identified. Most of the *YSL* genes were predicted to be plasma-membrane localized proteins, whose expressions were induced under Fe-limited conditions [59]. Our results revealed that TaYS1A localized in the plasma membrane of wheat protoplasts and *N. bethamiana* cells. Usually, many pathogenesis-related proteins are thought to be secreted into the apoplast to evade pathogenic infections by their defensive mechanism. Some of those proteins directly recognize and interact with the pathogens that exist in the plasma membrane [66,67]. Accordingly, we hypothesized that the presence of *TaYS1A* in the plasma-membrane might have a significant function in defense responses against pathogenic infection. However, the induction of SA might be required for the activation of *TaYS1A* in the plasma-membrane as endogenous SA treatment could significantly induce the expression of *TaYS1A*. SA has been confirmed to be synthesized by the plant in response to a diverse range of pathogens and it was also reported that SA is induced by *Pst* infection in wheat [8,11], suggesting the involvement of *TaYS1A* in the SA signaling pathway. Furthermore, we found that the expression of *TaYS1A* was significantly induced during wheat-*Pst* incompatible interaction. Thus, upon the infection of CYR23 and SA treatment in wheat leaves the expression of *TaYS1A* was significantly up-regulated, implying its crucial functions in wheat defense against *Pst*.

ROS have been proposed to play a key role in the establishment of plant defense followed by programmed cell death (PCD) [6,68] and are very effective against fungal infection. Specifically, H_2_O_2_ seems to elicit localized cell death and signals induction of pathogenesis-related genes and antioxidants in nearby plant cells during fungal infections [6,69]. Moreover, SA is a signaling molecule involved in basal and SAR with the activation of HR during infection caused by biotrophic pathogens [2,70]. HR occurs at or near the entry site after pathogen infection, followed by ROS production and SA accumulation. In addition to ROS production, SA can also inhibit ROS-scavenging by activating certain antioxidants, which ultimately contribute to the initiation of PCD at infection sites [8,71,72,73]. We used the VIGS method to explore the role of *TaYS1A* in the interaction between wheat and *Pst*. During the avirulent *Pst* CYR23 infections, *TaYS1A-*silenced plants were more susceptible, accompanied by decreased ROS accumulation and necrotic cell death, increased hyphal length, and expanded infection area, indicating that suppressing the expression of *TaYS1A* diminished wheat resistance to avirulent *Pst* infection. The transcript expression of ROS generating *TaNOX*, was also significantly reduced in *TaYS1A-*silenced plants infected with *Pst* CYR23, suggesting that *TaYS1A* regulated the wheat defense against *Pst* infection through the ROS-dependent pathway. Importantly, the increase of fungal biomass and the decrease in SA accumulation in *TaYS1A-*silenced plants infected with CYR23 further suggests that *TaYS1A* performs a positive regulatory function of wheat defense against *Pst* by modulating the SA signaling pathway. In contrast, the transcript level of ROS scavenging gene, *TaCAT*, was significantly upregulated after the silencing of *TaYS1A,* ultimately resulting in less ROS accumulation and promoting *Pst* infection. The reduction of ROS accumulation might have occurred due to the reduced SA accumulation in *TaYS1A*-silenced plants, which prevented inhibition of the ROS scavenging system. However, the mechanism by which *TaYS1A* modulates SA signaling or the ROS-dependent pathway to restrict *Pst* infection in wheat is unclear.

*NPR1* is the key positive SAR regulator and plays a crucial function in SA signal transduction to induce *PR* gene expression in *Arabidopsis* [74,75]. *NPR1* is a receptor of SA and transcriptional coregulator of SA-dependent defense-related gene activation [39,40]. The expression of *TaNH2*, an AtNPR3/4 orthologous gene, was induced by *Pst* infection and thereby performed a positive regulatory function in the resistance of wheat to *Pst* [11]. Our results revealed that TaYS1A directly interacted with TaNH2, and silencing of *TaYS1A* significantly reduced the transcript level of *TaNH2*. The transcript levels of *TaPR1* and *TaPR2* were also significantly diminished in *TaYS1A*-silenced leaves after infection with either CYR23 or CYR31. *AtYSL3* was reported to be induced by SA and required for pathogen defense in an *NPR1*-dependent pathway [38]. Thus, our results suggested that *TaYS1A* modulates SA signaling through the *TaNH2* activity that induces the transcription of SA-dependent defense-related genes and therefore increases wheat resistance against *Pst*.

In summary, our findings revealed that *TaYS1A* possibly functions as a positive wheat stripe rust resistance regulator by modulating the SA-signaling pathway via ROS-dependent signals. After pathogen invasion, plant biosynthesis SA serves as a part of pathogen defense, which may induce the transcription of *TaYS1A* to regulate SA-dependent signaling through ROS accumulation and defense-related gene expression. We further identified that TaYS1A directly interacts with TaNH2. *NPR1* genes are the central coregulator of defense-related gene activation in SA-signaling pathway and Fe acquisition and homeostasis could induce a burst of ROS during pathogen infection in plants [41,76]. We therefore hypothesize that the transcription of *TaYS1A* is activated by *TaNH2* by the induction of SA and the metal ion homeostasis function of *TaYS1A* performs a role in ROS accumulation leading to HR response in plant immunity against pathogen infection. Further studies are required to confirm our hypothesis. To our knowledge, this is the first report suggesting that a wheat *YSL* gene positively regulates the resistance against *Pst* infection, which expands our understanding toward the biological role of the *TaYS1A* gene, ranging from metal translocation to pathogen defense.

## Supplementary Materials

Supplementary materials can be found at https://www.mdpi.com/2073-4425/11/12/1452/s1. Figure S1: Phylogenetic analysis of *TaYS1A* and its homologs, Figure S2: Transcript profile of *TaYS1A* in different wheat tissues and hormone treatments, Figure S3: Multiple sequence alignment of the coding sequences of the *TaYS1A* copies, Figure S4: Growth of *Pst* virulent race CYR31 is reduced in *TaYS1A*-silenced plants, Figure S5: Host response of *TaYS1A*-silenced plants inoculated with *Pst* virulent race CRY31, Table S1: Primers used in this study.
