# Supplementary material for: TaYS1A, a Yellow Stripe-Like Transporter Gene, Is Required for Wheat Resistance to Puccinia striiformis f. sp. Tritici
Source: Genes (Basel). 2020 Dec 3;11(12):1452. doi: 10.3390/genes11121452 (PMC7761651; doi:10.3390/genes11121452)
Supplement: Supplementary file 1 [file genes-11-01452-s001.pdf]

SUPPLEMENTARY DATA

Article title: ***TaYS1A*, a Yellow stripe-like transporter gene, is required for wheat resistance to *Puccinia striiformis* f. sp. *tritici* (*Pst*)**

Authors: **Md Ashraful Islam, Jia Guo, Huan Peng, Shuxin Tian, Xingxuan Bai, Haochuan Zhu, Zhensheng Kang and Jun Guo**

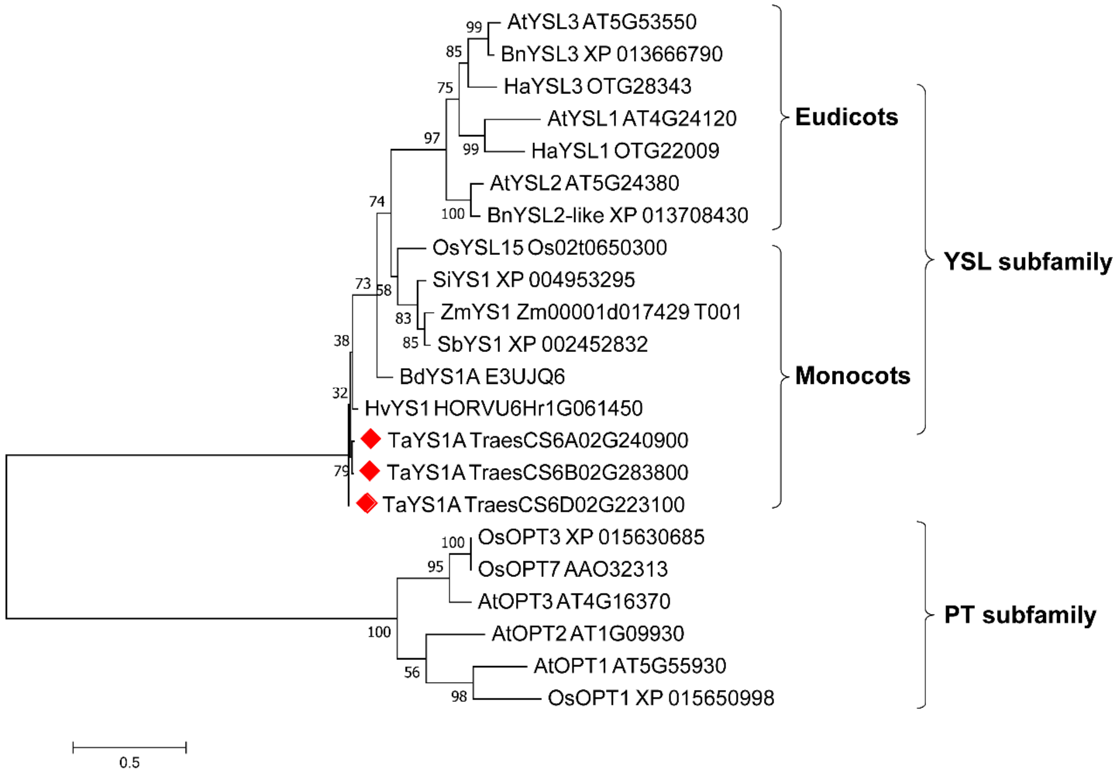

Figure S1 Phylogenetic analysis of *TaYS1A* and its homologs. MEGA7 software was used to generate the phylogenetic tree by the maximum likelihood method. The number above the internal branches indicates bootstrap values estimated based on 1000 replications. Branches are labeled with protein names and GenBank accession numbers. Ta, *Triticum aestivum;* Hv, *Hordeum vulgare;* Bd, *Brachypodium distachyon;* Os, *Oryza sativa;* Zm, *Zea mays;* Sb, *Sorghum bicolor;* Si, *Setaria italica;* At, *Arabidopsis thaliana;* Bn, *Brassica napus;* Ha, *Helianthus annuus.* YSL, yellow stripe-like transporters; PT, peptide transporters. PT was used as the outgroup.

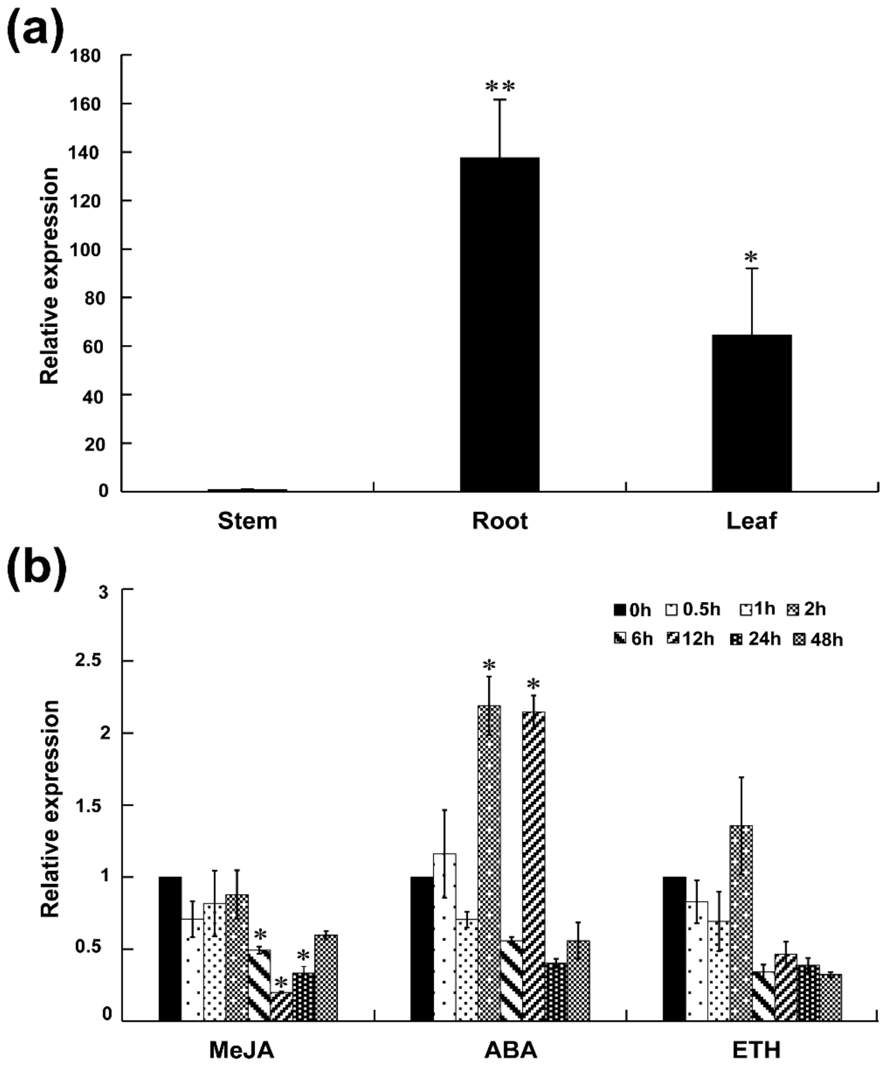

Figure S2 Transcript profile of *TaYS1A* in different wheat tissues and hormone treatments. **(a)** Samples were collected from leaves, stems and roots. **(b)** Wheat leaves treated with exogenous hormones: MeJA, methyl jasmonate; ABA, abscisic acid; ETH, ethylene. Relative transcript levels of *TaPho2* were calculated by the comparative threshold ($2^{-\Delta\Delta CT}$) method. The quantitative RT-PCR values were normalized with the transcript level of *TaEF-1α,* and presented as a fold changes relative to that in non-inoculated plants at time 0. The transcript level of *TaYS1A* in wheat leaves at time 0 was standardized as 1. Each data point represents means ± standard errors of three independent biological replications. Asterisks indicate $p < 0.05$ and double asterisks indicate $p < 0.01$ significant difference from the stem **(a)** and from 0 hpt **(b)** by Student's *t*-test.

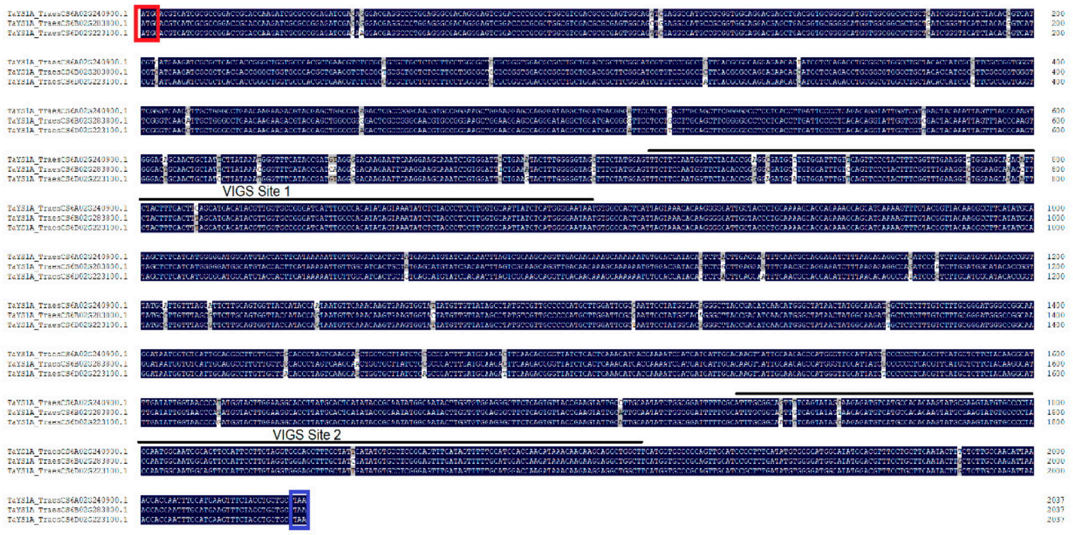

Figure S3 Multiple sequence alignment of the coding sequences of the *TaYS1A* copies. The coding sequences of *TaYS1A* copies in the genome database of wheat cv. Chinese Spring or cloned from wheat cv. Suwon11 were aligned by DNAMAN software. Red box: initiation codon (ATG), blue box: termination codon (TAA). The fragments for VIGS are indicated by overbars. VIGS site 1 to site 2 represent TaYS1A-1as to TaYS1A-2as, respectively.

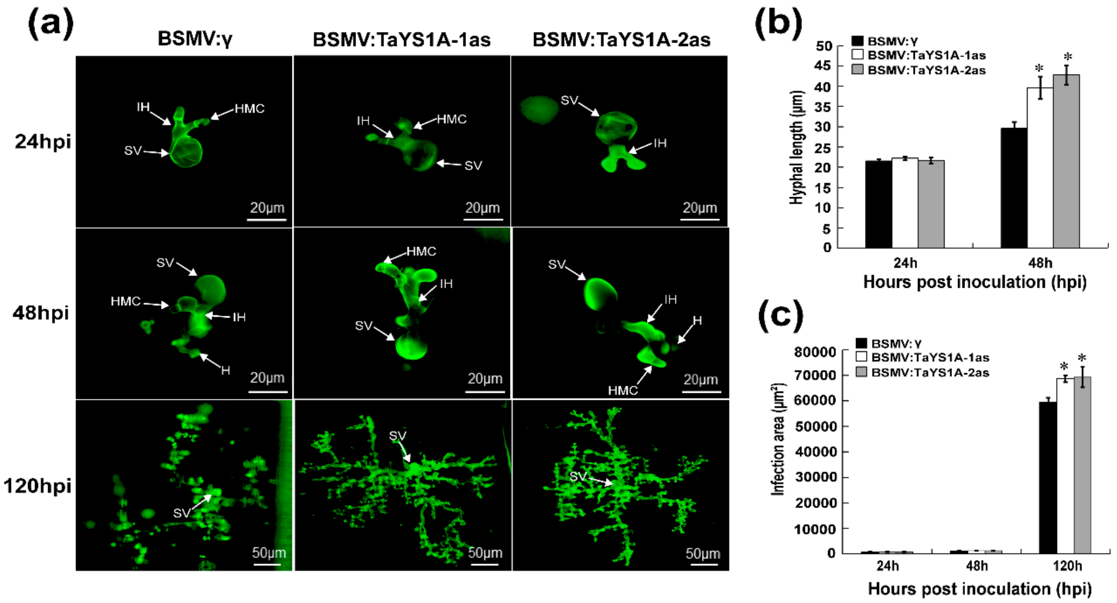

Figure S4 Growth of *Pst* virulent race CYR31 is reduced in *TaYS1A*-silenced plants. **(a)** The fungal structures were stained with wheat germ agglutinin (WGA) in wheat leaves inoculated with BSMV and *Pst* and observed under a fluorescence microscope. SV, sub-stomatal vesicle; HMC, haustorial mother cell; IH, infection hypha. H, haustoria. **(b)** Hyphal length of CYR31, which is the average distance from the junction of the sub-stomatal vesicle and the hypha to the tip of the hypha, was measured using DP-BSW software (units in μm). **(c)** Infection area of CYR31, the average of expanding hyphae, was calculated with DP-BSW software. All results were obtained from 50 infection sites and three biological replications

were performed. Asterisks indicated ($p < 0.05$) significant difference compared to that in BSMV:γ treatment plants at the same time points using Student's *t*-test.

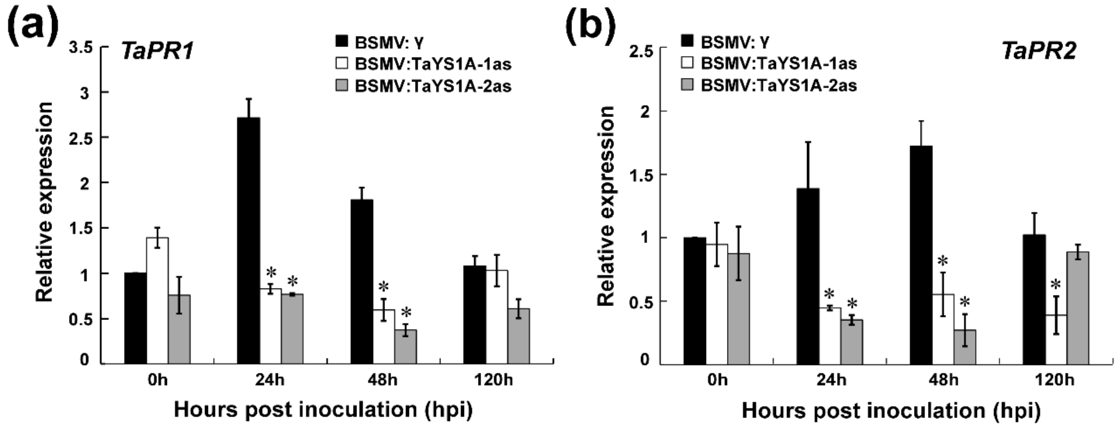

Figure S5. Host response of *TaYS1A*-silenced plants inoculated with *Pst* virulent race CRY31. **(a, b)** Relative transcript levels of *TaPR1* and *TaPR2* in *TaYS1A*-silenced plants inoculated with CYR31. The expression level was measured by qRT-PCR. The results were obtained from three biological replicates. The qRT-PCR values were normalized to those for *TaEF-1α,* and are presented as fold changes relative that in plants inoculated with BSMV:γ at time 0. The transcript level of genes in control plants at time 0 was standardized as 1. Data represent the mean of three biological replicates ± SE. Asterisks indicate ($p < 0.05$) significant differences between that in *TaYS1A*-silenced plants and control plants using Student's *t*-test.

Table S1. Primers used in this study.

Function	Name	Sequence 5' to 3'
Gene amplification	TaYS1A-S	ATGGACGTCATCGCGCCGGACCGCACC
	TaYS1A-AS	TTAGGCAGCAGGTAGAAACTTCATGC
qRT-PCR	TaYS1A-qRT-S	ATGACATACGTTGGTGCCGGGATGATTTGCCC
	TaYS1A-qRT-AS	CCCCCATGATGAGAGCTATGCATATGAAGGCC
	TaNH2-qRT-S	GGGAGGGGATGTTACCG
	TaNH2-qRT-AS	GAGCCTGCCCGTGTAGAG
	TaPR1-S	GAGAATGCAGACGCCCAAGC
	TaPR1-AS	CTGGAGCTTGCAGTCGTTGATC
	TaPR2-S	AGGATGTTGCTTCCATGTTTGCCG
	TaPR2-AS	AAGTAGATGCGCATGCCGTTGATG
	TaCAT-S	GCCCAAGTGCTCCCACCACAACA
	TaCAT-AS	TGAGGGTGCGGGAGGGGATG
	TaNOX-S	ATGTTCGGCAACTTGGTGACT
	TaNOX-AS	CGTCTGCTCTAAGAAGACCACTTTT
	TaEF-F	TGGTGTCATCAAGCCTGGTATGGT
	TaEF-R	ACTCATGGTGCATCTCAACGGACT
	PstEF-F	TTCGCCGTCCGTGATATGAGACAA
	PstEF-R	ATGCGTATCATGGTGGTGGAGTGA
Primer for VIGS	TaYS1A-V1-S	TAGCTAGCTGATTAATTAATTTCTTCCAATGGTTC TACACCG
	TaYS1A-V1-AS	TTGCTAGCTGAGCGGCCGCATTATTCCCCATGAG ATAATTGCA
	TaYS1A-V2-S	TAGCTAGCTGATTAATTAAATTTGCGGCAGTTGT CAGT
	TaYS1A-V2-AS	TTGCTAGCTGAGCGGCCGCAAGCCAGCCTCCTTC TTGT
Subcellular Localization	TaYS1A-163-S	TATCTCTAGAGGATCCATGGACGTCATCGCGCCG G
	TaYS1A-163-AS	TGCTCACCATGGATCCGGCAGCAGGTAGAAACT TCATGC
	TaYS1A-1302-S	CATGGTAGATCTGACTAGTATGGACGTCATCGCG CCG
	TaYS1A-1302-AS	GCCCTTGCTCACCATCCTAGGGGCAGCAGGTAG AAACTTCATGC
Primer for Y2H	TaYS1A-BD-S	TCAGAGGAGGACCTGCATATGATGGACGTCATC GCGCCG
	TaYS1A-BD-AS	TCGACGGATCCCCGGGAATTCGGCAGCAGGTAG AAACTTCATGC
	TaNH2-AD-S	TCCCCCGGGATGGAGCCGTCGTCGTCC
	TaNH2-AD-AS	CTAGTCTAGAGCTTCATCGCCGAGGATGA
